# Pralidoxime improves the hemodynamics and survival of rats with peritonitis-induced sepsis

**DOI:** 10.1371/journal.pone.0249794

**Published:** 2021-04-06

**Authors:** Najmiddin Mamadjonov, Yong Hun Jung, Kyung Woon Jeung, Hyoung Youn Lee, Byung Kook Lee, Chun Song Youn, In Seok Jeong, Tag Heo, Yong Il Min

**Affiliations:** 1 Department of Medical Science, Chonnam National University Graduate School, Gwangju, Republic of Korea; 2 Department of Emergency Medicine, Chonnam National University Hospital, Gwangju, Republic of Korea; 3 Department of Emergency Medicine, Chonnam National University Medical School, Gwangju, Republic of Korea; 4 Department of Emergency Medicine, Seoul St. Mary’s Hospital, The Catholic University of Korea, Seoul, Republic of Korea; 5 Department of Thoracic and Cardiovascular Surgery, Chonnam National University Hospital, Gwangju, Republic of Korea; University of Ljubljana, Medical faculty, SLOVENIA

## Abstract

Several studies have suggested that sympathetic overstimulation causes deleterious effects in septic shock. A previous study suggested that pralidoxime exerted a pressor effect through a mechanism unrelated to the sympathetic nervous system; this effect was buffered by the vasodepressor action of pralidoxime mediated through sympathoinhibition. In this study, we explored the effects of pralidoxime on hemodynamics and survival in rats with peritonitis-induced sepsis. This study consisted of two sub-studies: survival and hemodynamic studies. In the survival study, 66 rats, which survived for 10 hours after cecal ligation and puncture (CLP), randomly received saline placebo, pralidoxime, or norepinephrine treatment and were monitored for up to 24 hours. In the hemodynamic study, 44 rats were randomly assigned to sham, CLP-saline placebo, CLP-pralidoxime, or CLP-norepinephrine groups, and hemodynamic measurements were performed using a conductance catheter placed in the left ventricle. In the survival study, 6 (27.2%), 15 (68.1%), and 5 (22.7%) animals survived the entire 24-hour monitoring period in the saline, pralidoxime, and norepinephrine groups, respectively (log-rank test P = 0.006). In the hemodynamic study, pralidoxime but not norepinephrine increased end-diastolic volume (P <0.001), stroke volume (P = 0.002), cardiac output (P = 0.003), mean arterial pressure (P = 0.041), and stroke work (P <0.001). The pressor effect of norepinephrine was short-lived, such that by 60 minutes after the initiation of norepinephrine infusion, it no longer had any significant effect on mean arterial pressure. In addition, norepinephrine significantly increased heart rate (P <0.001) and the ratio of arterial elastance to ventricular end-systolic elastance (P = 0.010), but pralidoxime did not. In conclusion, pralidoxime improved the hemodynamics and 24-hour survival rate in rats with peritonitis-induced sepsis, but norepinephrine did not.

## Introduction

Sepsis is a systemic inflammatory response syndrome secondary to infection. Among sepsis-induced organ dysfunctions, cardiovascular dysfunction is a major contributor to the associated mortality [1, 2]. Norepinephrine is recommended as the vasopressor of choice for sepsis-induced hypotension [3]. However, responsiveness to this drug and other commonly used vasopressors is markedly reduced in septic shock [4–6]; patients who do not respond to vasopressors have extremely high mortality risks [7, 8]. Considering the high mortality of patients with septic shock [1, 2, 7, 8], novel therapeutics are required to improve the outcomes of these patients.

Pralidoxime (2-pyridine aldoxime methyl chloride) has been widely used to treat organophosphate poisoning because it can reactivate organophosphate-inhibited cholinesterase. Although several studies have reported its pressor effect [9–11], pralidoxime has never been used as a vasopressor agent in clinical practice. We previously reported that pralidoxime as an adjuvant to epinephrine increased aortic pressure during cardiopulmonary resuscitation in porcine models [12, 13]. We recently investigated the effects of adrenergic antagonists on the pressor action of pralidoxime in anesthetized normal rats to determine the involvement of the sympathetic nervous system in the pressor action of pralidoxime [14]. We found that the administration of 40 mg/kg of pralidoxime produced only a pressor response, while the administration of 200 mg/kg of pralidoxime produced an initial vasodepressor response followed by a delayed pressor response. Excluding sympathetic effects by means of adrenergic blockers (guanethidine and phentolamine) did not inhibit the pressor response of pralidoxime, but rather augmented it. Furthermore, in combination with the 200 mg/kg dose, the adrenergic blockers converted the initial vasodepressor response into a pressor response. These findings, taken together, indicated that pralidoxime did indeed have a dose-dependent vasodepressor action by mechanisms dependent on intact adrenergic receptor function, and that the pressor effect of pralidoxime was unrelated to the sympathetic nervous system. The latter’s vasodepressive action, mediated by inhibition of the sympathetic nervous system, acted to buffer the pralidoxime’s pressor action.

The activity of the sympathetic nervous system is increased in septic shock [15, 16]. Several studies suggest an association between sympathetic overstimulation and sepsis-induced cardiovascular dysfunction [17, 18]. Considering the non-adrenergic nature of the pressor effect of pralidoxime with its sympathoinhibitory effect, this drug may improve cardiovascular function in septic shock, ultimately leading to improved survival. In this study, we explored the effects of pralidoxime on hemodynamics and survival in rats with peritonitis-induced sepsis. We hypothesized that pralidoxime would improve hemodynamics and survival in this setting.

## Materials and methods

Male Wistar rats (300–330 g) were purchased from a commercial supplier (Samtako Bio Korea, Osan, Korea). They were housed two per cage for at least 7 days before experimentation in a temperature- and light-controlled room (22°C; 12-hour light/dark cycle), with free access to commercial feed and tap water. This study was approved by the Animal Care and Use Committee of Chonnam National University Hospital (CNUH IACUC-18003) and performed in accordance with the National Institutes of Health Guide for the Care and Use of Laboratory Animals. The investigators who conducted the experiments had completed an Institutional Animal Care and Use Committee training course on animal care and handling. All surgery was performed under isoflurane anesthesia, and all efforts were made to minimize suffering. This study comprised two sub-studies: one assessed the effects of the saline placebo, pralidoxime, and norepinephrine treatments on the survival rate (N = 84); the other assessed the hemodynamic effects of these treatments (N = 44).

### Surgical procedure for Cecal Ligation and Puncture (CLP)

A 2–3-cm ventral midline incision was made in each rat after inducing general anesthesia with 5% isoflurane. The cecum was exposed and ligated just distal to the ileocecal valve with a suture; it was then punctured through-and-through with an 18-gauge needle and compressed to extrude stool. The extruded stool was weighed using an electronic balance (CUX220H, CAS Korea, Seongnam, Korea). After returning the cecum and 300–400 mg of stool into the abdominal cavity, the incision was closed using sutures. Post-operative analgesia was provided with a subcutaneous injection of butorphanol (1 mg/kg), and the animals were returned to their cages. Animal health and behavior were monitored carefully every 30 minutes until the commencement of each study described below to determine if the rats met the pre-specified humane endpoints (unconsciousness, lack of reactivity, labored breathing, and cyanosis). Animals experiencing one or more of these endpoints were euthanized immediately by deep anesthesia with isoflurane.

### Survival study

Nine hours after the CLP, surviving animals were intubated using a 16-gauge catheter following the induction of anesthesia with 5% isoflurane and ventilated using a rodent ventilator with a 70/30 mixture of nitrous oxide and oxygen gas at a tidal volume of 6 ml/kg. The respiratory rate was titrated to achieve normocapnia. Throughout the experiment, the administered isoflurane was titrated to maintain adequate anesthesia (the dose of isoflurane required for this purpose was 1% in all animals), and butorphanol (1 mg/kg) was administered subcutaneously every 6 hours. A side stream end-tidal carbon dioxide sample line was placed in the ventilator circuit; the rat rectal temperature was maintained at 37–38°C using a heating pad. Twenty-four-gauge catheters were inserted into the left femoral and right jugular veins for the administration of norepinephrine and pralidoxime, respectively, and into the right femoral artery for arterial pressure monitoring. After baseline measurements were taken, imipenem (10 mg/kg, intravenously) and fluid for resuscitation (0.9% saline, 20 ml/kg intravenously over 20 minutes) were administered to mimic initial treatments in clinical practice.

An investigator, otherwise uninvolved with this study, assigned the animals to the control, pralidoxime, or norepinephrine groups using the sealed envelope method and prepared the prescribed drugs such that all other investigators remained blind to the group assignments. Each animal required one bag labeled as pralidoxime solution and one bag labeled as norepinephrine solution. In the pralidoxime group, the pralidoxime-labeled bags each contained 5 mg/ml pralidoxime solution, and the norepinephrine-labeled bags each contained 0.9% saline solution; in the norepinephrine group, the pralidoxime-labeled bags each contained 0.9% saline solution, and the norepinephrine-labeled bags each contained 0.08 mg/ml norepinephrine solution; in the control group, both bags contained 0.9% saline solution. Animals in all treatment groups also each received a separate 0.9% saline solution bag, labeled as such, for use in setting the overall infusion rate at 15 ml/kg/hour.

Ten hours after the CLP, we commenced experimental procedures. For each animal, regardless of group, pralidoxime-labeled solution was administered through the jugular venous catheter; norepinephrine-labeled solution was administered through the femoral venous catheter and was titrated up to a maximum of 1.5 ml/kg/hour to achieve the target mean arterial pressure (MAP) of ≥70 mmHg, in all groups, including those in which the norepinephrine-labeled bag contained saline. Norepinephrine-labeled solution was not administered if the MAP was 70 mmHg or higher. This approach was chosen to simulate clinical practice, in which norepinephrine dose is adjusted to predefined levels of arterial pressure. The correctly labeled saline was concurrently administered through the femoral venous catheter to ensure that despite any adjustments, the overall fluid infusion rate was 15 ml/kg/hour in all animals. The resultant actual treatments are listed below. Animals in the pralidoxime group were treated with an intravenous bolus (4 ml/kg, pralidoxime 20 mg/kg) followed by a continuous infusion of pralidoxime solution (4 ml/kg/hour, pralidoxime 20 mg/kg/hour), plus 0.9% saline solution (labeled as norepinephrine solution) titrated to achieve the target MAP, made up to the total rate of 15 ml/kg/hour with correctly-labeled saline. Adjustments made to the administration rate of the saline substituted for norepinephrine in this group varied within the same range as for the group receiving genuine norepinephrine. The pralidoxime dosing regimen was derived from that used in the treatment of organophosphate poisoning [19, 20]. Animals in the norepinephrine group were treated with an intravenous bolus (4 ml/kg) followed by a continuous infusion of 0.9% saline solution (4 ml/kg/hour, labeled as pralidoxime), plus norepinephrine solution titrated to achieve the target MAP, made up to the total rate of 15 ml/kg/hour with correctly-labeled saline. Animals in the control group received saline only, in the same protocol, i.e., an intravenous bolus (4 ml/kg) followed by a continuous infusion of 0.9% saline solution through the jugular venous catheter (labeled as pralidoxime solution, 4 ml/kg/hour) plus 0.9% saline solution (labeled as norepinephrine solution) titrated up to 1.5 ml/kg/hour to achieve the target MAP (as above), and another 0.9% saline solution, through the femoral venous catheter, adjusted to set the overall fluid infusion rate to 15 ml/kg/hour. Arterial pressure and heart rate were continuously monitored for up to 24 hours after the CLP. At the end of the 24-hour period or immediately upon the occurrence of signs of impending death (agonal breathing, cyanosis, and extreme bradycardia with hypotension), the animals were euthanized by exsanguination under general anesthesia.

### Hemodynamic study

Before the surgical procedure, animals were randomized to the sham or CLP group. The sham group animals underwent the same surgical procedure as the CLP group, except that the cecum was not ligated or punctured. Four hours after the CLP or sham operation, the animals were intubated, anesthetized, ventilated, and cannulated as described above. In addition, a 2.0 French pressure-volume conductance catheter (SPR-838, Millar Instruments, Houston, TX, USA) was inserted into the left ventricle (LV) through the right carotid artery to obtain the LV pressure-volume loops, and a 24-gauge catheter was inserted into the left jugular vein to monitor central venous pressure. Five hours after the CLP, the animals in the CLP group were randomly divided into the control, pralidoxime, or norepinephrine group. In the hemodynamic study, the time from CLP to study treatment was reduced to 5 hours to minimize the number of animals that died before the initiation of the study treatments, which were identical to those of the survival study, except for the norepinephrine infusion. The norepinephrine solution in the norepinephrine group (or 0.9% saline in the other groups) was administered at a fixed infusion rate of 1.5 ml/kg/hour (norepinephrine at 2 μg/kg/minute). In addition, norepinephrine infusion was started at 5 hours after the CLP irrespective of the MAP, to allow comparison of its effect with those of pralidoxime and saline placebo at the same timepoint. The animals were observed for 1 hour after study treatment initiation; they were then euthanized by exsanguination under general anesthesia.

The LV pressure-volume loops were analyzed using LabChart software (ADInstruments, Bella Vista, Australia) to measure end-diastolic volume (EDV), end-systolic volume, end-systolic pressure, arterial elastance (Ea), stroke volume, heart rate, cardiac output, ejection fraction, stroke work, and LV relaxation time constant. We did not perform preload manipulation through inferior vena cava compression for measurements of preload-independent parameters such as LV end-systolic elastance, since many rats experienced severe hemodynamic instability sustained even after release of the compression in our pilot experiments using the same model. Thus, LV end-systolic elastance estimated by single-beat method (Ees_sb_), calculated as the ratio of end-systolic pressure to end-systolic volume [21–23], was used as a surrogate for the LV end-systolic elastance. A ratio of Ea to Ees_sb_ was calculated to characterize ventriculo-arterial coupling. Systemic vascular resistance (SVR) was calculated as the difference between the MAP and central venous pressure divided by the cardiac output. In addition, rate pressure product, which reflects myocardial oxygen consumption, was calculated by multiplying systolic arterial pressure by heart rate [24]. Arterial pressure, central venous pressure (monitored from the jugular venous catheter), and hemodynamic parameters derived from the LV pressure-volume loops were sampled at baseline, 1-minute intervals for 5 minutes after study treatment initiation, and 15, 30, 45, and 60 minutes after study treatment initiation.

### Statistical analysis

The primary outcomes of the survival study were MAP and survival to 24 hours after CLP. The sample size for the survival study was calculated based on the MAP data of a pilot study (between-group variance = 31.443, within-group variance = 212.81, correlation among repeated measures = 0.6764), yielding a minimum requirement of 66 animals (22 per group) to reach an α of 0.05 and a power of 90%. We initially estimated that 94 animals would be needed for the survival study assuming 30% mortality before randomization. However, 84 rats were used, until there were 22 animals per group that survived up to the randomization. The sample size for the hemodynamic study was calculated based on the MAP data of the survival study. We estimated that 11 animals per group would be required to reach an α of 0.05 and a power of 80%. Continuous variables were tested for normality by using the Kolmogorov-Smirnov test and Shapiro-Wilk test. Normally distributed variables were summarized as mean ± standard deviation, whereas non-normally distributed variables were summarized as medians with interquartile ranges. One-way analysis of variance or Kruskal–Wallis test was performed to compare continuous variables among the three groups. Pairwise comparison with Bonferroni adjustment was performed for post-hoc analysis. In the hemodynamic study, the Mann-Whitney U test was used to compare continuous variables between the sham and CLP groups, and Wilcoxon signed rank test was used to compare the hemodynamic parameters before and after study treatment within the groups. Multiple linear mixed effect models with Bonferroni adjustments were used to analyze group effects and group-time interactions on serial hemodynamic measurements. Group, time, and their interaction were used as fixed effect covariates by controlling the random intercept and random slope of subjects. Survival curves were plotted using the Kaplan–Meier method and compared using the log-rank test, followed by pairwise comparison with Bonferroni adjustment. A two-tailed P value <0.05 was considered statistically significant. Data were analyzed using the R language version 3.3.3 (R Foundation for Statistical Computing, Vienna, Austria), T&F program version 3.0 (YooJin BioSoft, Goyang, Korea), and IBM SPSS Statistics for Windows version 22.0 (SPSS Inc., Chicago, IL, USA).

## Results

### Survival study

**Fig 1:**
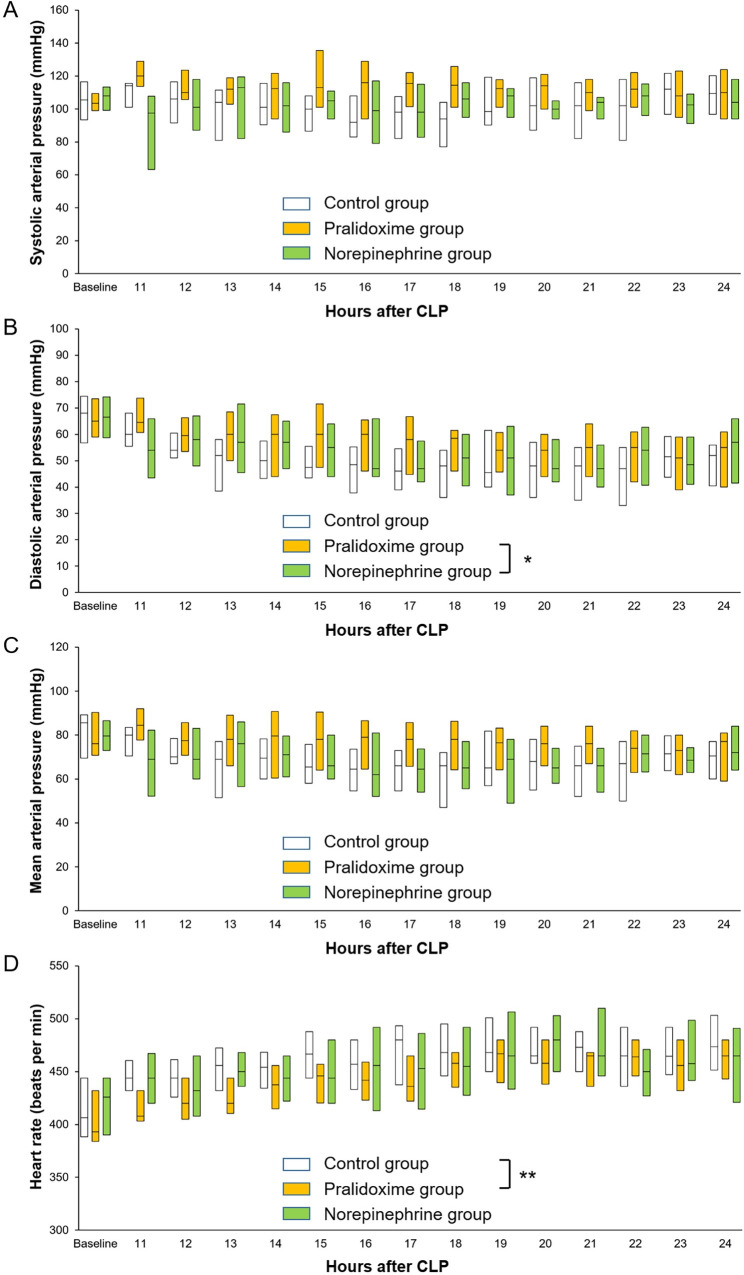
**Systolic arterial pressure (A), diastolic arterial pressure (B), mean arterial pressure (C), and heart rate (D) after the saline placebo, pralidoxime, and norepinephrine treatments.** Only the data of surviving animals at each time point are included. Data are medians (horizontal box dividers) with interquartile ranges (box size). CLP, cecal ligation and puncture. * P <0.05, group-time interaction and ** P <0.05, group effect using the linear mixed effect model.

**Fig 2:**
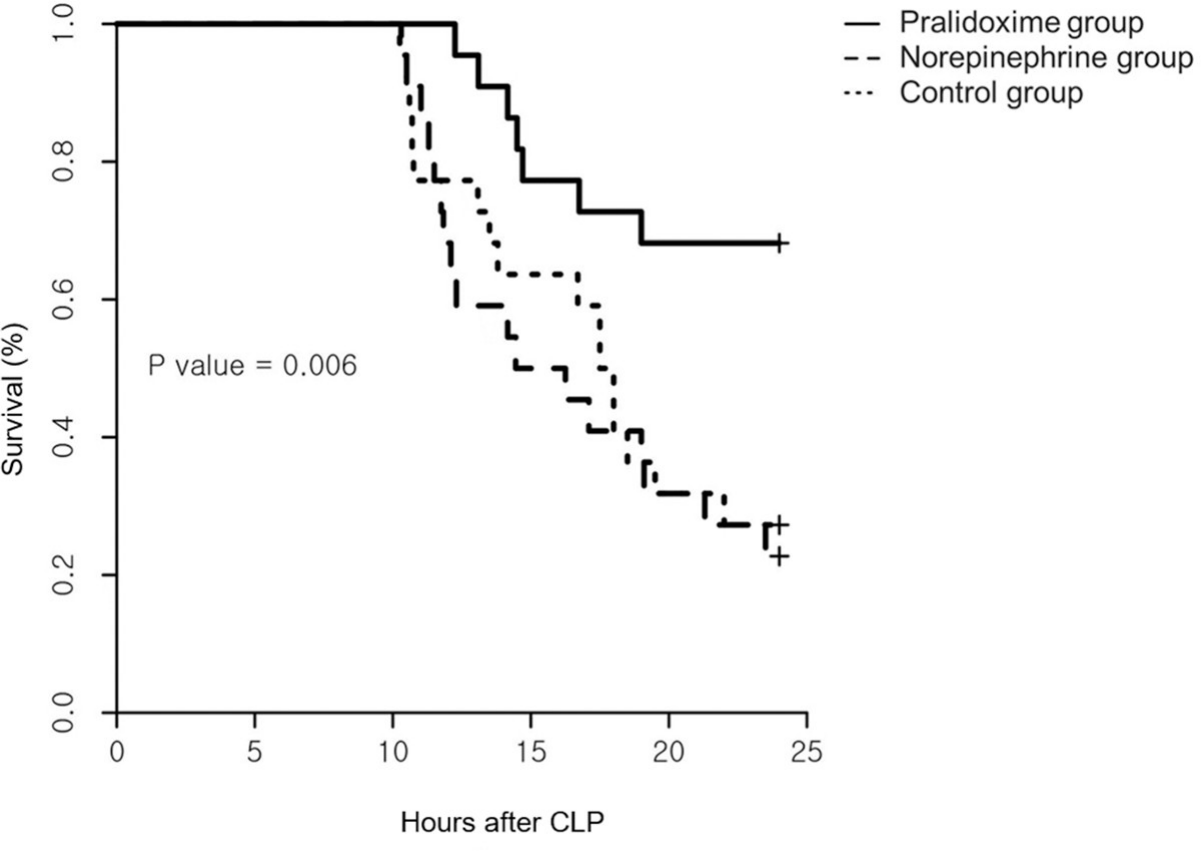
Survival curves following the saline placebo, pralidoxime, and norepinephrine treatments. Each treatment was initiated 10 hours after cecal ligation and puncture (CLP, N = 22 per group, P = 0.006 using the log-rank test).

Among the 84 rats that underwent CLP, 18 (21.4%) met the pre-specified humane endpoints and were euthanized before 9 hours after the CLP. The remaining 66 animals were randomized into three groups of 22 rats each. No inter-group differences were observed at baseline (Table 1). One animal in the norepinephrine group did not require norepinephrine administration until the end of the experiment, but data from this animal were included in the analysis. The norepinephrine group animals received 163 ± 138 μg norepinephrine for 218 (80–466) minutes (1.95 ± 0.52 μg/kg/minute). Fig 1 shows the arterial pressure and heart rate after randomization. The linear mixed effect model used to analyze the fixed effect of the pralidoxime group over that of the norepinephrine group revealed a significant group-time interaction for the diastolic arterial pressure (P = 0.019). Furthermore, the linear mixed effect model used to analyze the fixed effect of the pralidoxime group over that of the control group revealed a significant group effect for heart rate (P = 0.006). The pralidoxime group had the highest survival rate among the three groups (Fig 2). The result of the log-rank test revealed a significant difference in cumulative survival among the three groups (P = 0.006). Post-hoc analyses revealed higher survival in the pralidoxime group than in the norepinephrine group (P = 0.022).

**Table 1:** Baseline measurements.

Variable	Control group	Pralidoxime group	Norepinephrine group	P value^†^
	(N = 22)	(N = 22)	(N = 22)	
Weight (g)	310 (306–310)	310 (306–314)	310 (310–310)	0.675
Systolic arterial pressure (mmHg)	106 (94–116)	104 (99–107)	108 (101–112)	0.567
Diastolic arterial pressure (mmHg)	66 ± 10	66 ± 10	67 ± 12	0.968
Mean arterial pressure (mmHg)	81 ± 13	79 ± 12	80 ± 12	0.822
Heart rate (/min)	407 (392–444)	393 (384–429)	426 (394–442)	0.327
pH	7.438 ± 0.075	7.461 ± 0.095	7.438 ± 0.083	0.581
PaCO_2_ (mmHg)	34.9 ± 5.5	33.5 ± 4.4	35.2 ± 8.2	0.619
PaO_2_ (mmHg)	177.0 (151.5–195.0)	183.5 (158.5–197.8)	189.5 (148.3–199.5)	0.742
HCO_3_^-^ (mmol/l)	23.5 ± 3.1	24.0 ± 3.4	23.8 ± 4.3	0.912
Base excess (mmol/l)	0.6 (-2.8–2.3)	0.9 (-1.3–2.2)	0.3 (-1.1–1.8)	0.824
SaO_2_ (%)	100 (99–100)	100 (100–100)	100 (99–100)	0.624
Hemoglobin (g/dl)	15.1 (13.6–16.1)	14.9 (13.2–15.8)	15.4 (14.1–16.1)	0.804

Data are the means ± standard deviation or medians with interquartile ranges.

^†^ P values were calculated using one-way analysis of variance or the Kruskal–Wallis test. PaCO_2_, partial pressure of carbon dioxide; PaO_2_, partial pressure of oxygen; HCO_3_^-^, bicarbonate; SaO_2_, oxygen saturation.

### Hemodynamic study

**Fig 3:**
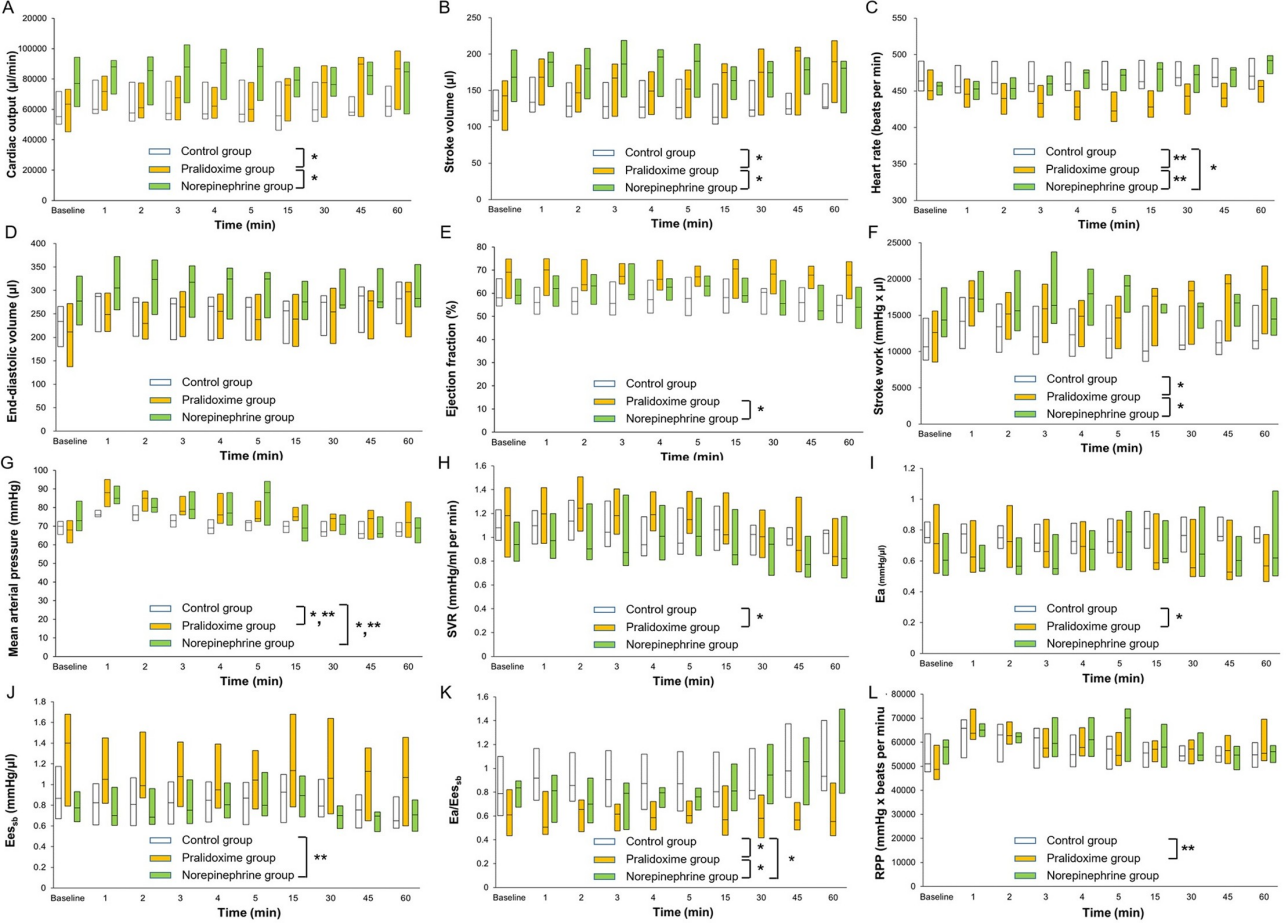
**Cardiac output (A), stroke volume (B), heart rate (C), end-diastolic volume (D), ejection fraction (E), stroke work (F), mean arterial pressure (G), systemic vascular resistance (H), arterial elastance (I), left ventricular end-systolic elastance (J), ratio of arterial elastance to left ventricular end-systolic elastance (K), and rate pressure product (L) between baseline and 60 minutes after study treatment initiation.** Data are medians (horizontal box dividers) with interquartile ranges (box size). SVR, systemic vascular resistance; Ea, arterial elastance; Ees_sb_, left ventricular end-systolic elastance; RPP, rate pressure product. * P <0.05, group-time interaction and ** P <0.05, group effect using the linear mixed effect model.

**Fig 4:**
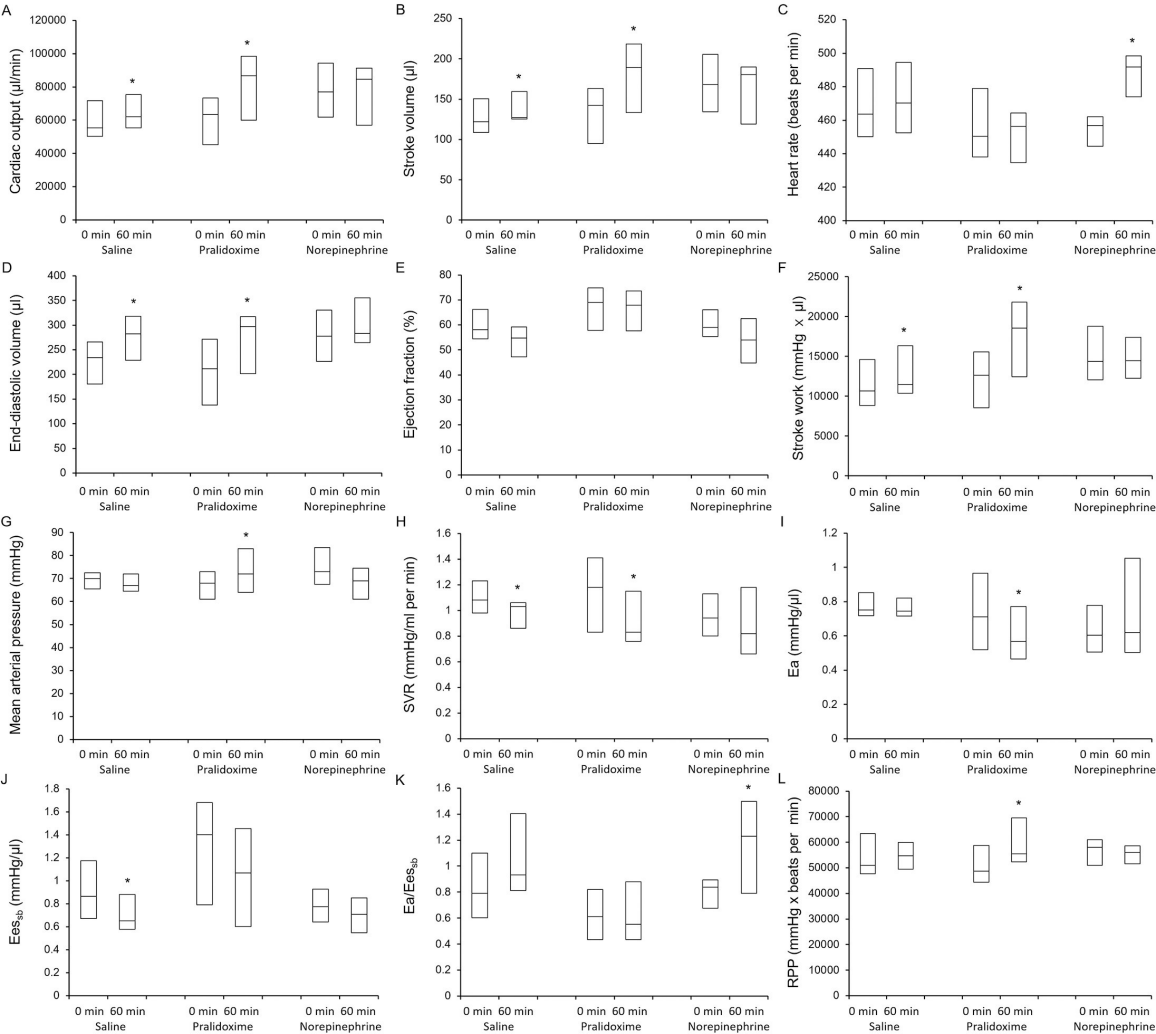
Effects of saline placebo, pralidoxime, and norepinephrine on cardiac output (A), stroke volume (B), heart rate (C), end-diastolic volume (D), ejection fraction (E), stroke work (F), mean arterial pressure (G), systemic vascular resistance (H), arterial elastance (I), left ventricular end-systolic elastance (J), ratio of arterial elastance to left ventricular end-systolic elastance (K), and rate pressure product (L). Data are medians (horizontal box dividers) with interquartile ranges (box size). * P <0.05 versus 0 minutes using Wilcoxon signed rank test. SVR, systemic vascular resistance; Ea, arterial elastance; Ees_sb_, left ventricular end-systolic elastance; RPP, rate pressure product.

All the 44 rats that underwent CLP or the sham operation survived until the end of the experiments. At baseline, the animals that underwent CLP had higher Ea/Ees_sb_ (P <0.001) and lower arterial pressure (P <0.001), LV ejection fraction (P = 0.005), LV relaxation time constant (P = 0.001), Ees_sb_ (P = 0.011), and rate pressure product (P <0.001) than the animals in the sham group (Table 2), but no significant differences in the hemodynamic parameters were observed among the three CLP groups (Table 3). Fig 3 shows the hemodynamic data from baseline to 60 minutes after study treatment initiation. The linear mixed effect model used to analyze the fixed effect of the pralidoxime group over that of the control group revealed significant group effects for heart rate (P <0.001) and rate pressure product (P = 0.001), significant group-time interactions for cardiac output (P <0.001), stroke volume (P = 0.002), stroke work (P = 0.012), SVR (P = 0.021), Ea (P = 0.030), and Ea/Ees_sb_ (P = 0.001), and a significant group effect and a group-time interaction for MAP (P <0.001 and P = 0.023, respectively). The linear mixed effect model used to analyze the fixed effect of the pralidoxime group over that of the norepinephrine group revealed a significant group effect for heart rate (P <0.001) and significant group-time interactions for cardiac output (P = 0.007), stroke volume (P = 0.004), LV ejection fraction (P = 0.003), stroke work (P = 0.001), and Ea/Ees_sb_ (P <0.001). The linear mixed effect model used to analyze the fixed effect of the norepinephrine group over that of the control group revealed a significant group effect for Ees_sb_ (P = 0.005), significant group-time interactions for heart rate (P = 0.003) and Ea/Ees_sb_ (P = 0.018), and a significant group effect and a group-time interaction for MAP (P = 0.040 and P = 0.009, respectively). The effects of the saline placebo, pralidoxime, and norepinephrine treatments on the hemodynamic parameters are summarized in Fig 4. At 60 minutes after the saline placebo treatment, the EDV (P = 0.005), stroke volume (P = 0.024), stroke work (P = 0.032), and cardiac output (P = 0.032) were significantly higher than their baseline values, whereas the SVR (P = 0.003) and Ees_sb_ (P = 0.014) were significantly lower. The pralidoxime group showed a significantly higher EDV (P <0.001), stroke volume (P = 0.002), cardiac output (P = 0.003), MAP (P = 0.041), stroke work (P <0.001), and rate pressure product (P = 0.024) and a significantly lower SVR (P = 0.014) and Ea (P = 0.003) 60 minutes after pralidoxime treatment than at baseline. The norepinephrine group showed no significant changes in hemodynamic parameters 60 minutes after the norepinephrine treatment compared to the baseline values, except for the heart rate and Ea/Ees_sb_; the heart rate (P <0.001) and Ea/Ees_sb_ (P = 0.010) were significantly higher.

**Table 2:** Effects of cecal ligation and puncture on hemodynamic parameters at baseline.

Variable	Sham group (N = 11)	CLP group (N = 33)	P value^†^
Weight (g)	315 (310–318)	310 (310–320)	0.773
Systolic arterial pressure (mmHg)	153 (136–165)	113 (103–131)	<0.001
Diastolic arterial pressure (mmHg)	68 (60–81)	47 (43–55)	<0.001
Mean arterial pressure (mmHg)	102 (83–109)	70 (65–74)	<0.001
End-tidal carbon dioxide (mmHg)	38 (37–42)	39 (37–41)	0.702
End-diastolic volume (μl)	238 (165–265)	253 (184–284)	0.593
Stroke volume (μl)	162 (140–199)	138 (102–168)	0.295
Heart rate (beats per min)	481 (439–498)	457 (441–477)	0.406
Cardiac output (μl/min)	75,020 (65,850–85,125)	63,820 (49,780–82,530)	0.283
Ejection fraction (%)	71 (66–80)	62 (56–69)	0.005
Stroke work (mmHg × μl)	17,350 (13,735–18,620)	12,620 (8,602–16,830)	0.078
Systemic vascular resistance (mmHg/ml per min)	1.24 (0.92–1.66)	1.08 (0.85–1.30)^‡^	0.315
Left ventricular relaxation time constant (ms)	7.68 (6.70–8.68)	6.18 (5.48–6.50)	0.001
Ea (mmHg/μl)	0.619 (0.525–0.864)	0.736 (0.548–0.920)	0.810
Ees_sb_ (mmHg/μl)	1.711 (0.940–1.852)	0.854 (0.658–1.401)	0.011
Ea/Ees_sb_	0.484 (0.316–0.563)	0.776 (0.572–0.925)	<0.001
Rate pressure product (mmHg × beats per min)	72,009 (60,297–78,114)	52,969 (47,736–61,690)	<0.001

Data are the medians with interquartile ranges.

^†^ P values were calculated using the Mann-Whitney U test.

^‡^ Systemic vascular resistance was not obtained in one animal because of technical issues. CLP, cecal ligation and puncture; Ea, arterial elastance; Ees_sb_, left ventricular end-systolic elastance.

**Table 3:** Hemodynamic measurements at baseline in the control, pralidoxime, and norepinephrine groups.

Variable	Control group	Pralidoxime group	Norepinephrine group	P value^†^
	(N = 11)	(N = 11)	(N = 11)	
Systolic arterial pressure (mmHg)	113 (103–131)	105 (102–128)	127 (111–137)	0.268
Diastolic arterial pressure (mmHg)	46 (43–50)	46 (40–56)	55 (48–60)	0.092
Mean arterial pressure (mmHg)	70 (66–73)	68 (61–73)	73 (68–84)	0.152
End-tidal carbon dioxide (mmHg)	40 (38–41)	39 (37–41)	39 (36–40)	0.683
End-diastolic volume (μl)	234 (180–266)	212 (137–272)	277 (226–331)	0.235
Stroke volume (μl)	122 (109–151)	143 (95–163)	168 (135–206)	0.311
Heart rate (beats per min)	464 (450–491)	451 (438–479)	457 (445–462)	0.545
Cardiac output (μl/min)	55,360 (50,335–71,820)	63,450 (45,310–73,280)	77,120 (61,785–94,355)	0.409
Ejection fraction (%)	58 (54–66)	69 (58–75)	59 (55–66)	0.209
Stroke work (mmHg × μl)	10,650 (8,837–14,605)	12,620 (8,565–15,580)	14,360 (12,040–18,805)	0.482
Systemic vascular resistance (mmHg/ml per min)	1.08 (0.98–1.23)	1.18 (0.83–1.42)	0.94 (0.80–1.13)^‡^	0.741
Left ventricular relaxation time constant (ms)	6.29 (5.73–6.65)	6.06 (5.32–6.66)	6.11 (5.43–6.44)	0.767
Ea (mmHg/μl)	0.751 (0.717–0.853)	0.712 (0.519–0.966)	0.605 (0.506–0.779)	0.514
Ees_sb_ (mmHg/μl)	0.865 (0.671–1.175)	1.401 (0.791–1.681)	0.775 (0.641–0.930)	0.364
Ea/Ees_sb_	0.790 (0.603–1.100)	0.610 (0.435–0.820)	0.837 (0.674–0.894)	0.257
Rate pressure product (mmHg × beats per min)	51,005 (47,703–63,465)	48,654 (44,434–58,722)	58,014 (50,949–60,995)	0.545

Data are the medians with interquartile ranges.

^†^ P values were calculated using the Kruskal–Wallis test.

^‡^ Systemic vascular resistance was not obtained in one animal because of technical issues. Ea, arterial elastance; Ees_sb_, left ventricular end-systolic elastance.

## Discussion

To the best of our knowledge, this is the first study investigating the use of pralidoxime as a primary vasopressor in the treatment of septic shock. In this study, pralidoxime improved the EDV, stroke volume, cardiac output, MAP, and stroke work, whereas norepinephrine failed to improve these hemodynamic parameters. Importantly, pralidoxime treatment resulted in the best survival rate among the three groups.

Several studies have suggested that adrenergic vasopressors have deleterious effects, including tachycardia, myocardial cell damage, and deterioration of myocardial function [18, 25–27]. In a study that examined the effect of norepinephrine infusion on cardiac performance using a rat model of peritonitis-induced sepsis [26], norepinephrine infusion resulted in significant decreases in the LV ejection fraction and fractional shortening. In a study of 112 intensive care unit patients with cardiovascular failure, the extent and duration of catecholamine therapy were independently associated with the occurrence of adverse cardiac events including tachyarrhythmia, myocardial cell damage, and reduction of systemic blood flow [18]. In addition, studies have indicated that the attenuation of sympathetic activity improves cardiovascular function and survival in septic shock [28–30]. Considering these studies suggesting the harmful effects of sympathetic overstimulation in septic shock [17, 18, 28–30], the non-adrenergic nature of the pressor effect of pralidoxime with its sympathoinhibitory effect (reported in our previous study [14]) could have led to the superiority of the hemodynamic and survival benefits of this drug over those of norepinephrine in the present study.

In this study, the pressor effect of pralidoxime was mediated by its action on stroke volume and cardiac output rather than on SVR. The increase in stroke volume after pralidoxime treatment seemed largely driven by its negative chronotropic effect. In both the survival and hemodynamic studies, pralidoxime treatment produced the lowest heart rate among the three groups. There was a significant group effect for heart rate in both studies. This was consistent with our previous study where pralidoxime significantly decreased the heart rate compared with saline placebo in anesthetized normal rats [13]. In the present study, the increase in stroke volume after pralidoxime administration was not accompanied by an increase in the LV ejection fraction but rather by an increase in EDV and a decrease in heart rate (although this result was not statistically significant; see Fig 4). This finding indicates that the increase in stroke volume after pralidoxime treatment was a result of the improved LV diastolic filling caused by an increase in the time for diastolic filling. Importantly, despite the decrease in heart rate, the increased stroke volume after pralidoxime administration significantly increased the cardiac output. As shown in Fig 3, the stroke volume and cardiac output markedly increased over time in the pralidoxime group relative to the control group, and the linear mixed effect models including pralidoxime and control groups showed significant group-time interactions for both stroke volume and cardiac output. The increase in cardiac output could have contributed to the survival benefit conferred by pralidoxime. These hemodynamic effects of pralidoxime resembled those reported for β blockers in multiple previous studies [28, 31]. In a study that examined the effects of esmolol, a short-acting β blocker, on cardiovascular function in rats with peritonitis-induced sepsis [28], the esmolol-induced reduction in heart rate was accompanied by a significant increase in stroke volume without any associated improvement in LV ejection fraction. In addition, the animals treated with esmolol showed better survival than those that were not. In a randomized clinical trial that compared esmolol infusion titrated to maintain the heart rate between 80 beats/minute and 94 beats/minute with standard treatment for patients with septic shock [31], the esmolol infusion resulted in lower heart rate, higher stroke volume and LV stroke work, and lower 28-day mortality than the standard treatment. In contrast, the marked increase in heart rate after norepinephrine treatment observed in the hemodynamic study could be detrimental. Several studies have suggested that tachycardia is significantly associated with poor outcomes in patients with septic shock [27, 32, 33].

Consistently with previous results suggesting ventriculo-arterial decoupling in septic shock [34, 35], the Ea/Ees_sb_ was markedly increased in our septic animals as compared to that in our sham group animals. In a study that investigated ventriculo-arterial coupling in 25 septic and 25 non-septic patients [34], the ratio of peripheral Ea to LV end-systolic elastance (estimated by using the single beat method) was significantly higher in septic patients than in non-septic patients (1.81 [1.49–2.03] vs. 1.07 [0.95–1.18], P = 0.01). In our study, the Ea/Ees_sb_ fell after pralidoxime treatment, as shown in Fig 4, although this did not reach statistical significance. Meanwhile, norepinephrine increased the Ea and decreased the Ees_sb_ (albeit non-significantly), which resulted in the significant increase in Ea/Ees_sb_. As shown in Fig 3, the Ea/Ees_sb_ increased over time in the norepinephrine group, while it remained close to the baseline values in the pralidoxime group. These findings indicate that norepinephrine significantly impaired ventriculo-arterial coupling in our model, while no such adverse effect followed pralidoxime treatment.

The rate pressure product has been demonstrated to correlate with myocardial oxygen consumption [24]. In our hemodynamic study, the rate pressure product was significantly decreased in the septic animals as compared to that in the sham group animals. This finding is consistent with several previous studies [36, 37]. In studies using rat models of sepsis, Mathieu et al. and Wang et al. reported significant reductions in rate pressure product after CLP and lipopolysaccharide challenge, respectively [36, 37]. In our study, the rate pressure product was increased after the pralidoxime treatment, but was not increased after the norepinephrine or saline placebo treatment. Our findings suggest that pralidoxime improved cardiac function, and thereby increased cardiac work and myocardial oxygen consumption, all of which were abnormally depressed by sepsis.

Previous studies have reported impairments to several action mechanisms of catecholamine in septic shock, including systemic downregulation of adrenoceptor gene expression and inactivation of adrenoceptors [38, 39]. Studies have also reported that peroxynitrite, a reactive oxidant produced from nitric oxide and superoxide, contributes to the reduced responsiveness to catecholamine in septic shock by deactivating catecholamines [40, 41]. Consistently with these studies [38–41], norepinephrine failed to improve hemodynamic parameters and survival rate in this study. Instead, it adversely affected hemodynamic parameters. As shown in Fig 3, heart rate and Ea/Ees_sb_ increased over time in the norepinephrine group relative to the control group, and the mixed linear effect models including norepinephrine and control groups showed significant group-time interactions for heart rate and Ea/Ees_sb_. The MAP was transiently increased after norepinephrine administration, resulting in the significant group effect and group-time interaction in the linear mixed effect model including norepinephrine and control groups. However, MAP returned to baseline values within 60 minutes. In our previous study [13], an intravenous bolus administration of 20 mg/kg pralidoxime increased the MAP by approximately 5 mmHg in anesthetized normal rats. In this study, the increase in MAP after pralidoxime treatment in the hemodynamic study (4 [2.5–6.5] mmHg) was similar, suggesting that the action mechanism of pralidoxime, which remains to be determined but is non-adrenergic in nature, may not be impaired in septic shock. These findings are also in line with previous studies reporting reduced responsiveness to catecholamine in the presence of relatively preserved responsiveness to non-catecholamine vasopressors in septic shock [5, 42, 43]. In a study that investigated arterial pressure responses to norepinephrine, vasopressin, and F-180, a selective V_1_ receptor agonist, in rats [5], Barrett et al. reported that the arterial pressure responses to norepinephrine were markedly reduced in rats with fecal peritonitis compared with sham-surgery controls, while the responses to vasopressin and F-180 were relatively preserved in rats with fecal peritonitis. In a study that investigated the effects of norepinephrine, vasopressin, terlipressin, vasopressin plus norepinephrine, and terlipressin plus norepinephrine in rats subjected to CLP [42], vasopressin and terlipressin either alone or in combination with norepinephrine improved MAP and survival while norepinephrine alone did not. Further studies are needed to determine the effects of sepsis on the pressor effect of pralidoxime.

The pressor effect of norepinephrine was short-lived in the present study, resulting in no significant effect on MAP 60 minutes after the initiation of norepinephrine infusion. Previous studies showed conflicting results of the hemodynamic effects of norepinephrine in septic shock, which depended on the experimental conditions [35, 42, 44]. The inability of norepinephrine to improve arterial pressure in our study might be related to the sepsis severity of our model, which was high (as evidenced by the 72.7% mortality rate in the control group). We ligated the cecum just distal to the ileocecal valve (rather than at a more distal portion), punctured it through-and-through with an 18-gauge needle (rather than a single puncture with a smaller gauge needle), and extruded a large amount of stool to achieve a high-severity sepsis model to allow the monitoring of treatment effects. Alternatively, the inability of norepinephrine to improve arterial pressure in our study might be attributable to the time of evaluation in our study. Norepinephrine (0.25–4 μg/kg/min) administered between 10 and 14 hours after CLP did not increase arterial pressure in a study using a rat model of peritonitis-induced sepsis [42], whereas, in studies using rat models of peritonitis-induced sepsis where norepinephrine was administered after more than 24 hours [5, 45], it increased arterial pressure.

This study has several limitations. First, it was conducted in rats, and the findings are not directly extrapolatable to humans. Second, we used a high-severity sepsis model, which could have exaggerated the reduced responsiveness to norepinephrine. In addition, the volume of intravenous fluid bolus administered for initial resuscitation (20 ml/kg) was lower than that recommended in the Survival Sepsis Campaign guidelines (30 ml/kg) [46, 47]. Unlike typical clinical settings, where the intravenous fluid infusion rate is adjusted according to intravascular volume status, our intravenous fluid infusion following initial resuscitation was performed at a weight-based fixed rate. The intravenous fluid administration might thus have been insufficient, and this might have blunted the hemodynamic effects of study drugs. Third, the average infusion rate of norepinephrine in the survival study was close to or higher than those used in multiple previous studies using rat models [35, 44, 45], but higher norepinephrine infusion rate might have led to different results. In addition, norepinephrine was administered at a fixed timepoint irrespective of MAP in our hemodynamic study, which might limit the translatability of its findings to human septic shock. Fourth, our study focused on macrocirculatory hemodynamics. Previous studies have reported the dissociation between macro- and microcirculation in septic shock [48, 49]. Further studies are needed to examine the microcirculatory effects of pralidoxime. Fifth, this study employed a pralidoxime dosing regimen used in the treatment of organophosphate poisoning. Further studies are required to identify the optimal dosing regimen in septic shock. Sixth, the anesthetic and analgesic agents used in this study might have affected the study groups differently, in terms of both hemodynamic parameters and survival. Isoflurane and butorphanol have been shown to affect hemodynamics and cardiac function, as well as norepinephrine kinetics [50–53]. An experimental study also reported that norepinephrine abolished the anti-inflammatory effects of isoflurane in rats with endotoxemia [54]. In addition, mechanical ventilation itself could have affected our results. Mechanical ventilation modulates hemodynamics and cardiac output. An experimental study reported that mechanical ventilation also significantly affected the duration of survival in rats with sepsis [55]. Thus, our findings should be verified in unanesthetized spontaneously breathing animals. Seventh, the time from CLP to study treatment differed between the hemodynamic and the survival studies. Thus, the results of hemodynamic study might have been different from those that could have been obtained in the setting of the survival study, in which the time from CLP to study treatment was longer. Eighth, we could not unravel the mechanism underlying the hemodynamic benefit of pralidoxime observed in the present study. While the mechanism of the antidotal effect of pralidoxime is well known, limited information is available on the mechanism underlying its hemodynamic effect [9–11, 14]. To our knowledge, the effects of pralidoxime on the mechanisms responsible for sepsis-induced cardiovascular dysfunction and vascular hyporeactivity to catecholamine vasopressors, including nitric oxide overproduction and adrenoceptor downregulation in response to high circulating catecholamine levels, have not yet been investigated. In addition, the sympathoinhibitory effect of pralidoxime has been reported only in our previous study [14]. Hence, further studies investigating these factors in detail are needed. Lastly, the Ees_sb_ might not have reflected the LV end-systolic elastance accurately, as it was calculated on the assumption that the volume-axis intercept of end-systolic pressure-volume relationship was negligible.

## Conclusions

In conclusion, pralidoxime but not norepinephrine improved the hemodynamics and 24-hour survival rate in a rat model of peritonitis-induced sepsis. In view of these results, pralidoxime treatment in septic shock deserves further evaluation.

## Supporting information

S1 Data Raw data. (XLSX)

S1 File ARRIVE guidelines checklist. (DOC)

## References

[1] Shankar-Hari M, Phillips GS, Levy ML, Seymour CW, Liu VX, Deutschman CS, et al. Developing a New Definition and Assessing New Clinical Criteria for Septic Shock: For the Third International Consensus Definitions for Sepsis and Septic Shock (Sepsis-3). JAMA. 2016;315: 775–787. 10.1001/jama.2016.0289 26903336 PMC4910392

[2] Landesberg G, Gilon D, Meroz Y, Georgieva M, Levin PD, Goodman S, et al. Diastolic dysfunction and mortality in severe sepsis and septic shock. Eur Heart J. 2012;33: 895–903. 10.1093/eurheartj/ehr351 21911341 PMC3345552

[3] Rhodes A, Evans LE, Alhazzani W, Levy MM, Antonelli M, Ferrer R, et al. Surviving Sepsis Campaign: International Guidelines for Management of Sepsis and Septic Shock: 2016. Intensive Care Med. 2017;43: 304–377. 10.1007/s00134-017-4683-6 28101605

[4] Chen SJ, Li SY, Shih CC, Liao MH, Wu CC. NO contributes to abnormal vascular calcium regulation and reactivity induced by peritonitis-associated septic shock in rats. Shock. 2010;33: 473–478. 10.1097/SHK.0b013e3181bea334 19749606

[5] Barrett LK, Orie NN, Taylor V, Stidwill RP, Clapp LH, Singer M. Differential effects of vasopressin and norepinephrine on vascular reactivity in a long-term rodent model of sepsis. Crit Care Med. 2007;35: 2337–2343. 10.1097/01.ccm.0000281861.72907.17 17944022

[6] Hollenberg SM, Tangora JJ, Piotrowski MJ, Easington C, Parrillo JE. Impaired microvascular vasoconstrictive responses to vasopressin in septic rats. Crit Care Med. 1997;25: 869–873. 10.1097/00003246-199705000-00025 9187609

[7] Brand DA, Patrick PA, Berger JT, Ibrahim M, Matela A, Upadhyay S, et al. Intensity of Vasopressor Therapy for Septic Shock and the Risk of In-Hospital Death. J Pain Symptom Manage. 2017;53: 938–943. 28062334 10.1016/j.jpainsymman.2016.12.333

[8] Conrad M, Perez P, Thivilier C, Levy B. Early prediction of norepinephrine dependency and refractory septic shock with a multimodal approach of vascular failure. J Crit Care. 2015;30: 739–743. 10.1016/j.jcrc.2015.03.029 25900257

[9] Zarro VJ, Dipalma JR. The sympathomimetic effects of 2-pyridine aldoxime methylchloride (2-PAM CL). J Pharmacol Exp Ther. 1965;147: 153–160. 14259492

[10] Stavinoha WB, Hinshaw LB, Smith PW, Rieger JA Jr. Cardiovascular effects of 2-pyridine aldoxime methylchloride (pralidoxime and blood pressure). Arch Int Pharmacodyn Ther. 1970;187: 52–65. 5480142

[11] Carrier GO, Peters T, Bishop VS. Alteration in calcium metabolism as a mechanism for pyridine aldoxime methochloride (2-PAM) cardiac action in rabbit atria. J Pharmacol Exp Ther. 1975;193: 218–231. 1133766

[12] Jung YH, Ryu DH, Jeung KW, Na J, Lee DH, Lee BK, et al. Effect of pralidoxime on coronary perfusion pressure during cardiopulmonary resuscitation in a pig model. Clin Exp Emerg Med. 2019;6: 204–211. 10.15441/ceem.18.036 31036784 PMC6774006

[13] Jung YH, Lee HY, Jeung KW, Lee BK, Youn CS, Yun SW, et al. Pralidoxime administered during cardiopulmonary resuscitation facilitates successful resuscitation in a pig model of cardiac arrest. Clin Exp Pharmacol Physiol. 2020;47: 236–246. 10.1111/1440-1681.13198 31631356

[14] Jung YH, Mamadjonov N, Lee HY, Jeung KW, Lee BK, Youn CS, et al. Effects of Different Doses of Pralidoxime Administered During Cardiopulmonary Resuscitation and the Role of α-Adrenergic Receptors in Its Pressor Action. J Am Heart Assoc. 2020;9: e015076. 10.1161/JAHA.119.015076 32070203 PMC7335542

[15] Ramchandra R, Wan L, Hood SG, Frithiof R, Bellomo R, May CN. Septic shock induces distinct changes in sympathetic nerve activity to the heart and kidney in conscious sheep. Am J Physiol Regul Integr Comp Physiol. 2009;297: R1247–R1253. 10.1152/ajpregu.00437.2009 19726712

[16] Benedict CR, Rose JA. Arterial norepinephrine changes in patients with septic shock. Circ Shock. 1992;38: 165–172. 1292880

[17] Schmittinger CA, Dünser MW, Torgersen C, Luckner G, Lorenz I, Schmid S, et al. Histologic pathologies of the myocardium in septic shock: a prospective observational study. Shock. 2013;39: 329–335. 10.1097/SHK.0b013e318289376b 23376953

[18] Schmittinger CA, Torgersen C, Luckner G, Schröder DC, Lorenz I, Dünser MW. Adverse cardiac events during catecholamine vasopressor therapy: a prospective observational study. Intensive Care Med. 2012;38: 950–958. 10.1007/s00134-012-2531-2 22527060

[19] Schexnayder S, James LP, Kearns GL, Farrar HC. The pharmacokinetics of continuous infusion pralidoxime in children with organophosphate poisoning. J Toxicol Clin Toxicol. 1998;36: 549–555. 9776957

[20] Pralidoxime: Drug information. [cited 15 January 2021]. In: UpToDate 19.3 [Internet]. Waltham, Massachusetts: UpToDate. [about 2 screens]. Available from: https://somepomed.org/articulos/contents/mobipreview.htm?39/41/40605.

[21] Monge García MI, Santos A. Understanding ventriculo-arterial coupling. Ann Transl Med. 2020;8: 795. 10.21037/atm.2020.04.10 32647720 PMC7333110

[22] Bombardini T, Costantino MF, Sicari R, Ciampi Q, Pratali L, Picano E. End-systolic elastance and ventricular-arterial coupling reserve predict cardiac events in patients with negative stress echocardiography. Biomed Res Int. 2013;2013: 235194. 10.1155/2013/235194 24024185 PMC3760182

[23] Maurer MS, Sackner-Bernstein JD, El-Khoury Rumbarger L, Yushak M, King DL, Burkhoff D. Mechanisms underlying improvements in ejection fraction with carvedilol in heart failure. Circ Heart Fail. 2009;2: 189–196. 10.1161/CIRCHEARTFAILURE.108.806240 19808339

[24] Gobel FL, Norstrom LA, Nelson RR, Jorgensen CR, Wang Y. The rate-pressure product as an index of myocardial oxygen consumption during exercise in patients with angina pectoris. Circulation. 1978;57: 549–556. 10.1161/01.cir.57.3.549 624164

[25] Communal C, Singh K, Pimentel DR, Colucci WS. Norepinephrine stimulates apoptosis in adult rat ventricular myocytes by activation of the beta-adrenergic pathway. Circulation. 1998;98: 1329–1334. 10.1161/01.cir.98.13.1329 9751683

[26] Chopra M, Das P, Golden H, Dostal DE, Watson LE, Sharma AC. Norepinephrine induces systolic failure and inhibits antiapoptotic genes in a polymicrobial septic rat model. Life Sci. 2010;87: 672–678. 10.1016/j.lfs.2010.09.029 20933523

[27] Domizi R, Calcinaro S, Harris S, Beilstein C, Boerma C, Chiche J, et al. Relationship between norepinephrine dose, tachycardia and outcome in septic shock: A multicentre evaluation. J Crit Care. 2020;57: 185–190. 10.1016/j.jcrc.2020.02.014 32171905

[28] Kimmoun A, Louis H, Al Kattani N, Delemazure J, Dessales N, Wei C, et al. β1-Adrenergic Inhibition Improves Cardiac and Vascular Function in Experimental Septic Shock. Crit Care Med. 2015;43: e332–e340. 10.1097/CCM.0000000000001078 25962080

[29] Ackland GL, Yao ST, Rudiger A, Dyson A, Stidwill R, Poputnikov D, et al. Cardioprotection, attenuated systemic inflammation, and survival benefit of beta1-adrenoceptor blockade in severe sepsis in rats. Crit Care Med. 2010;38: 388–394. 10.1097/CCM.0b013e3181c03dfa 19829100

[30] Wilson J, Higgins D, Hutting H, Serkova N, Baird C, Khailova L, et al. Early propranolol treatment induces lung heme-oxygenase-1, attenuates metabolic dysfunction, and improves survival following experimental sepsis. Crit Care. 2013;17: R195. 10.1186/cc12889 24020447 PMC4056775

[31] Morelli A, Ertmer C, Westphal M, Rehberg S, Kampmeier T, Ligges S, et al. Effect of Heart Rate Control With Esmolol on Hemodynamic and Clinical Outcomes in Patients With Septic Shock: A Randomized Clinical Trial. JAMA. 2013;310: 1683–1691. 10.1001/jama.2013.278477 24108526

[32] Parker MM, Shelhamer JH, Natanson C, Alling DW, Parrillo JE. Serial cardiovascular variables in survivors and nonsurvivors of human septic shock: heart rate as an early predictor of prognosis. Crit Care Med. 1987;15: 923–929. 10.1097/00003246-198710000-00006 3652707

[33] Leibovici L, Gafter-Gvili A, Paul M, Almanasreh N, Tacconelli E, Andreassen S, et al. Relative tachycardia in patients with sepsis: an independent risk factor for mortality. QJM. 2007;100: 629–634. 10.1093/qjmed/hcm074 17846061

[34] Guarracino F, Ferro B, Morelli A, Bertini P, Baldassarri R, Pinsky MR. Ventriculoarterial decoupling in human septic shock. Crit Care. 2014;18: R80. 10.1186/cc13842 24762124 PMC4056562

[35] Ducrocq N, Kimmoun A, Furmaniuk A, Hekalo Z, Maskali F, Poussier S, et al. Comparison of equipressor doses of norepinephrine, epinephrine, and phenylephrine on septic myocardial dysfunction. Anesthesiology. 2012;116: 1083–1091. 10.1097/ALN.0b013e31824f9669 22407285

[36] Wang YL, Lam KK, Cheng PY, Kung CW, Chen SY, Chao CC, et al. The cardioprotective effect of hypertonic saline is associated with inhibitory effect on macrophage migration inhibitory factor in sepsis. Biomed Res Int. 2013;2013: 201614. 10.1155/2013/201614 24371817 PMC3858963

[37] Mathieu C, Desrois M, Kober F, Lalevée N, Lan C, Fourny N, et al. Sex-Mediated Response to the Beta-Blocker Landiolol in Sepsis: An Experimental, Randomized Study. Crit Care Med. 2018;46: e684–e691. 10.1097/CCM.0000000000003146 29634521

[38] Bucher M, Kees F, Taeger K, Kurtz A. Cytokines down-regulate alpha1-adrenergic receptor expression during endotoxemia. Crit Care Med. 2003;31: 566–571. 10.1097/01.CCM.0000048621.36569.69 12576967

[39] Takakura K, Taniguchi T, Muramatsu I, Takeuchi K, Fukuda S. Modification of alpha1 -adrenoceptors by peroxynitrite as a possible mechanism of systemic hypotension in sepsis. Crit Care Med. 2002;30: 894–899. 10.1097/00003246-200204000-00030 11940765

[40] Takakura K, Xiaohong W, Takeuchi K, Yasuda Y, Fukuda S. Deactivation of norepinephrine by peroxynitrite as a new pathogenesis in the hypotension of septic shock. Anesthesiology. 2003;98: 928–934. 10.1097/00000542-200304000-00020 12657855

[41] Takakura K, Xiaohong W, Takeuchi K, Fukuda S. Peroxynitrite decreases dopamine’s vasoconstrictive activity. Anesth Analg. 2003;97: 1492–1496. 10.1213/01.ane.0000082248.30437.0b 14570672

[42] Xiao X, Zhu Y, Zhen D, Chen XM, Yue W, Liu L, et al. Beneficial and side effects of arginine vasopressin and terlipressin for septic shock. J Surg Res. 2015;195: 568–579. 10.1016/j.jss.2015.02.022 25769491

[43] Bennett T, Mahajan RP, March JE, Kemp PA, Gardiner SM. Regional and temporal changes in cardiovascular responses to norepinephrine and vasopressin during continuous infusion of lipopolysaccharide in conscious rats. Br J Anaesth. 2004;93: 400–407. 10.1093/bja/aeh214 15220167

[44] Fries M, Ince C, Rossaint R, Bleilevens C, Bickenbach J, Rex S, et al. Levosimendan but not norepinephrine improves microvascular oxygenation during experimental septic shock. Crit Care Med. 2008;36: 1886–1891. 10.1097/CCM.0b013e31817cede9 18496356

[45] Blet A, Deniau B, Geven C, Sadoune M, Caillard A, Kounde PR, et al. Adrecizumab, a non-neutralizing anti-adrenomedullin antibody, improves haemodynamics and attenuates myocardial oxidative stress in septic rats. Intensive Care Med Exp. 2019;7: 25. 10.1186/s40635-019-0255-0 31093784 PMC6520420

[46] Dellinger RP, Levy MM, Rhodes A, Annane D, Gerlach H, Opal SM, et al. Surviving sepsis campaign: international guidelines for management of severe sepsis and septic shock: 2012. Crit Care Med. 2013;41: 580–637. 10.1097/CCM.0b013e31827e83af 23353941

[47] Levy MM, Evans LE, Rhodes A. The Surviving Sepsis Campaign Bundle: 2018 Update. Crit Care Med. 2018;46: 997–1000. 10.1097/CCM.0000000000003119 29767636

[48] De Backer D, Creteur J, Preiser JC, Dubois MJ, Vincent JL. Microvascular blood flow is altered in patients with sepsis. Am J Respir Crit Care Med. 2002;166: 98–104. 10.1164/rccm.200109-016oc 12091178

[49] Fries M, Weil MH, Sun S, Huang L, Fang X, Cammarata G, et al. Increases in tissue Pco2 during circulatory shock reflect selective decreases in capillary blood flow. Crit Care Med. 2006;34: 446–452. 10.1097/01.ccm.0000196205.23674.23 16424727

[50] Sano Y, Ito S, Yoneda M, Nagasawa K, Matsuura N, Yamada Y, et al. Effects of various types of anesthesia on hemodynamics, cardiac function, and glucose and lipid metabolism in rats. Am J Physiol Heart Circ Physiol. 2016;311: H1360–H1366. 10.1152/ajpheart.00181.2016 27694213

[51] Deegan R, He HB, Wood AJ, Wood M. Effect of enflurane and isoflurane on norepinephrine kinetics: a new approach to assessment of sympathetic function during anesthesia. Anesth Analg. 1993;77: 49–54. 10.1213/00000539-199307000-00010 8317746

[52] Muldoon S, Otto J, Freas W, Watson RL. The effects of morphine, nalbuphine, and butorphanol on adrenergic function in canine saphenous veins. Anesth Analg. 1983;62: 21–28. 6129820

[53] dos Santos PS, Nunes N, de Souza AP, de Rezende ML, Nishimori CT, de Paula DP, et al. Hemodynamic effects of butorphanol in desflurane-anesthetized dogs. Vet Anaesth Analg. 2011;38: 467–474. 10.1111/j.1467-2995.2011.00644.x 21831052

[54] Hofstetter C, Boost KA, Hoegl S, Flondor M, Scheller B, Muhl H, et al. Norepinephrine and vasopressin counteract anti-inflammatory effects of isoflurane in endotoxemic rats. Int J Mol Med. 2007;20: 597–604. 17786293

[55] Tang W, Pakula JL, Weil MH, Noc M, Fukui M, Bisera J. Adrenergic vasopressor agents and mechanical ventilation for the treatment of experimental septic shock. Crit Care Med. 1996;24: 125–130. 10.1097/00003246-199601000-00021 8565517

